# Building a decoder of perceptual decisions from microsaccades and pupil size

**DOI:** 10.3389/fpsyg.2022.942859

**Published:** 2022-09-13

**Authors:** Ryohei Nakayama, Jean-Baptiste Bardin, Ai Koizumi, Isamu Motoyoshi, Kaoru Amano

**Affiliations:** ^1^Department of Psychology, The University of Tokyo, Tokyo, Japan; ^2^Center for Information and Neural Networks (CiNet), National Institute of Information and Communications Technology, Osaka, Japan; ^3^École Polytechnique Fédérale de Lausanne, Lausanne, Switzerland; ^4^Sony Computer Science Laboratories, Inc., Tokyo, Japan; ^5^Department of Life Sciences, The University of Tokyo, Tokyo, Japan; ^6^Graduate School of Information Science and Technology, The University of Tokyo, Tokyo, Japan

**Keywords:** visual awareness, adaptation-induced blindness, classifier, microsaccade, pupil size

## Abstract

Many studies have reported neural correlates of visual awareness across several brain regions, including the sensory, parietal, and frontal areas. In most of these studies, participants were instructed to explicitly report their perceptual experience through a button press or verbal report. It is conceivable, however, that explicit reporting itself may trigger specific neural responses that can confound the direct examination of the neural correlates of visual awareness. This suggests the need to assess visual awareness without explicit reporting. One way to achieve this is to develop a technique to predict the visual awareness of participants based on their peripheral responses. Here, we used eye movements and pupil sizes to decode trial-by-trial changes in the awareness of a stimulus whose visibility was deteriorated due to adaptation-induced blindness (AIB). In the experiment, participants judged whether they perceived a target stimulus and rated the confidence they had in their perceptual judgment, while their eye movements and pupil sizes were recorded. We found that not only perceptual decision but also perceptual confidence can be separately decoded from the eye movement and pupil size. We discuss the potential of this technique with regard to assessing visual awareness in future neuroimaging experiments.

## Introduction

One of the ultimate goals of neuroscience is to reveal how conscious experience emerges from neural activities. One promising approach for achieving this goal is to identify the neural correlates of consciousness (NCC; Koch, 2004), which is defined as the minimal set of neuronal mechanisms that is jointly sufficient for any specific conscious percept. Because the studies on NCC require the assessment of participants’ perceptual experiences, researchers typically ask participants to explicitly report their perceptual decisions *via* a button press or verbal report. However, such explicit reporting may lead to an overestimation of the neural correlates because the reporting itself could induce some neural responses (Tsuchiya et al., 2015). Indeed, Frässle et al. (2014) have shown that prefrontal activity reflects the responses related to the reporting rather than those related to the perceptual switch itself. Additionally, an electroencephalogram (EEG) experiment utilizing an inattentional blindness paradigm revealed that the P3 component of event-related potentials as well as gamma-band activity emerge only when participants performed a task while paying attention to the stimulus (Pitts et al., 2014); this is consistent with the possibility that these neural responses correlate with reporting rather than perceptual awareness *per se*. Although it is debatable whether the neural activities are completely absent without the reporting or are present only to a lesser, undetectable level, these studies suggest that conventional subjective report paradigm may not be the best way to isolate NCC. Therefore, it is valuable to develop a reliable no-report paradigm where we can predict the differences in perceptual decisions regarding stimuli without explicit reporting (e.g., Hesse and Tsao, 2020; Kapoor et al., 2022).

A conventional procedure for comparing the neural responses between different perceptual states involves utilizing bistable perceptual phenomena such as binocular rivalry (Alais and Blake, 2005; Tong et al., 2006) or motion-induced blindness (MIB; Bonneh et al., 2001). Alternatively, by adjusting the stimulus onset asynchrony between the target and mask stimuli in metacontrast masking, we can create a situation where the same target stimulus is perceived in some trials but not in other trials (Breitmeyer and Ogmen, 2000). Although these procedures are very useful for inducing different percepts of the stimulus while keeping its physical properties constant, the perception of target stimuli is suppressed by other competing stimuli, such as stimuli in the other eye for the binocular rivalry, motion stimuli for the MIB, and mask stimuli for metacontrast masking. Therefore, it would be difficult to use these conventional procedures to distinguish the neural responses to target stimuli and those to competing stimuli. One effective technique to resolve this issue is to use a phenomenon called adaptation-induced blindness (AIB; Motoyoshi and Hayakawa, 2010), where adaptation to a moving stimulus proactively reduces the visibility of a subsequently presented target stimulus. As AIB does not require the simultaneous presentation of competing stimuli and reportedly has little impact on early visual processes (Motoyoshi and Hayakawa, 2010), it enables us to isolate the neural responses related to the perceptual decisions for a target stimulus, which would be ideal for studying NCC.

Although some other no-report techniques have been previously suggested, the use of those methods is limited to the assessment of visual awareness for specific types of stimuli. In binocular rivalry, a perceived stimulus can be predicted by the optokinetic nystagmus (OKN)—pursuit and saccade eye movements alternately elicited to track field motion—if the stimuli presented in each eye are moving in opposite directions (Frässle et al., 2014; Kapoor et al., 2022). Although this technique is useful for moving stimuli, our perceptual awareness often fluctuates with static stimuli in various visual tasks as well as in daily situations, which would not induce OKN. Therefore, it is valuable to develop a technique to predict the perceptual decisions for a target stimulus without relying on OKN. In this study, we examine whether the peripheral responses, namely microsaccades and pupil size, can reliably predict moment-to-moment changes in the perception of a static stimulus presented along with AIB.

Microsaccades have been shown to produce weak transient signals during fixation (Martinez-Conde et al., 2004), counteracting Troxler fading (Martinez-Conde et al., 2006) and MIB (Hsieh and Tse, 2009). In addition, one study has shown that microsaccades tend to be more frequent and directed toward the peripheral target when it was visible (Cui et al., 2009). Because these studies convergently suggest that microsaccades are related to perceptual awareness, here, we hypothesized that they may entail information regarding the prediction of perceptual awareness in AIB. Additionally, we used pupil size as a secondary peripheral response to predict perceptual awareness, as studies have demonstrated that pupil dilatation was time-locked to the appearance of an attended peripheral cue (Smallwood et al., 2011; Kang et al., 2014) and was representative of attentional processing (Wierda et al., 2012). In particular, pupil size has been used to decode binary decisions on a single-trial basis [e.g., intention (Bednarik et al., 2012); object identification (Jangraw et al., 2014); cognitive state (Medathati et al., 2020); letter identification (Strauch et al., 2021)]. Pupil dilation has also been associated with certainty or confidence in decision making [e.g., memory, language, reasoning, perception (Beatty, 1982 for a review); categorical judgments (Brunyé and Gardony, 2017); attentional selection (Geng et al., 2015); inference learning (Nassar et al., 2012)]. While microsaccades and pupil size covary to some extent in various cognitive contexts (Privitera et al., 2010, 2014; Wang et al., 2017; Krejtz et al., 2018; Raj et al., 2019) and during microstimulation of the superior colliculus (Wang and Munoz, 2021), some differential modulations have also been reported (Strauch et al., 2018; Dalmaso et al., 2020; Krejtz et al., 2020; Salvi et al., 2020; Wang et al., 2020). Thus, these parameters may possibly provide at least partly distinct information for building a decoder of perceptual decisions.

As an initial step, to establish no-report paradigms, the present study validated whether perceptual decisions and confidence regarding target presence could be decoded based on the microsaccade rate and pupil size. The results indicated that the perceptual decision (detection of a target stimulus) can be decoded from a subset of simple features of the microsaccades and pupil size. The results further showed that perceptual confidence can be also decoded from eye movements and pupil size significantly above chance, although using a different subset of features. We will discuss how this technique can be potentially improved and applied for neuroimaging experiments to study NCC with a no-report paradigm.

## Results

**FIGURE 1:**
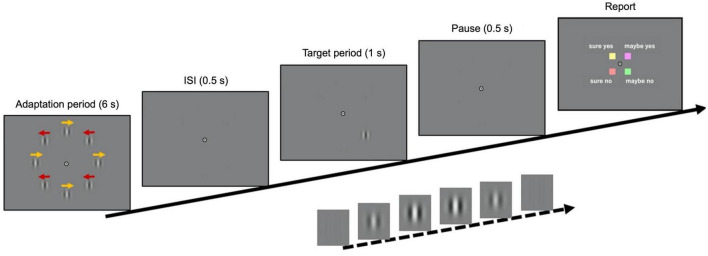
Time course of visual stimuli. The adaptation stimulus, composed of eight drifting Gabor patches, was presented for 60 s (the first trial) or 6 s (all other trials). After an inter-stimulus interval of 0.5 s, a target stimulus was presented in 80% of the trials for 1 s (either at the top-left or bottom-right positions in 65% of the trials, and at the other positions in 15%). In the remaining 20% of the trials, the target was absent. When a target was presented, its contrast was filtered with a temporal Gaussian window to reach its maximum value 0.5 s after the onset. The maximum contrast in the main experiment was determined in a calibration experiment using a staircase method. Finally, after a pause of 0.5 s, the response key assignment was displayed until the participant responded.

In a given experimental trial, eight stimuli with moving internal patterns were presented during an adaptation period of 6 s (Figure 1; see the “Materials and methods” section for details). After an inter-stimulus interval (ISI) of 0.5 s, a static target stimulus, in which the contrast was filtered through a temporal Gaussian window, was presented for 1 s. It was displayed either at the top-left or bottom-right position (positions of interest) in 65% of the trials and at the other six locations in 15% of the trials. The remaining 20% of the trials were catch trials, where the target stimulus was absent. The participants were instructed to maintain fixation on a bullseye at the center of the monitor and report *via* button press if they perceived the target stimulus, along with their confidence level regarding the same (i.e., “sure yes,” “sure no,” “maybe yes,” or “maybe no”). The experiment was composed of a calibration block followed by four main blocks of 43 trials each. In the calibration experiment, the contrast threshold of the target stimulus was estimated using a staircase method. The target contrast in the main experiment was fixed at the threshold level {32.2 ± 8.8% [standard error (SE)]}. The gaze position, velocity, instantaneous acceleration, and pupil size of both eyes were monitored during the task.

### Behavioral responses

**FIGURE 2:**
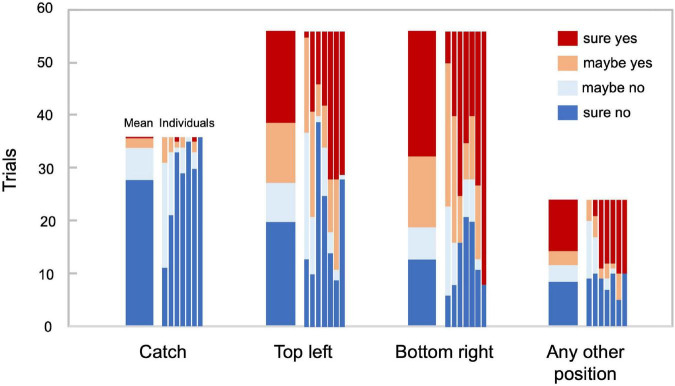
Behavioral responses. Trial count for each behavioral response in 36 catch trials, 56 trials in which the target stimulus was presented at the top left and the bottom right, respectively, and the remaining 24 trials, in which the target stimulus was presented at any other position. The adjacent bars correspond to average and individual responses, respectively (*N* = 7).

Figure 2 illustrates the response distributions for the catch trials and those in which a target stimulus was presented in the positions of interest and in any other position, respectively. In the catch trials, no participant reported seeing a target in more than five out of 36 trials, ensuring their proper engagement in the task. The target stimulus was reported to be visible in the top-left position in 51.6% of the trials, while it was visible in the bottom-right position in 66.3% of the trials. Although the number of visible and invisible stimulus trials was not fully balanced, in the main analysis, we did not equate the number of trials by resampling because such an imbalance might reveal information about perceptual decisions. We calculated the accuracy and area under the receiver operating characteristic (ROC) curve (AUC), which is suitable for unbalanced data. We did not use eye movement directions as inputs for the decoder so that the difference in the hit rates across positions would not bias the decision boundary of the decoder.

**FIGURE 3:**
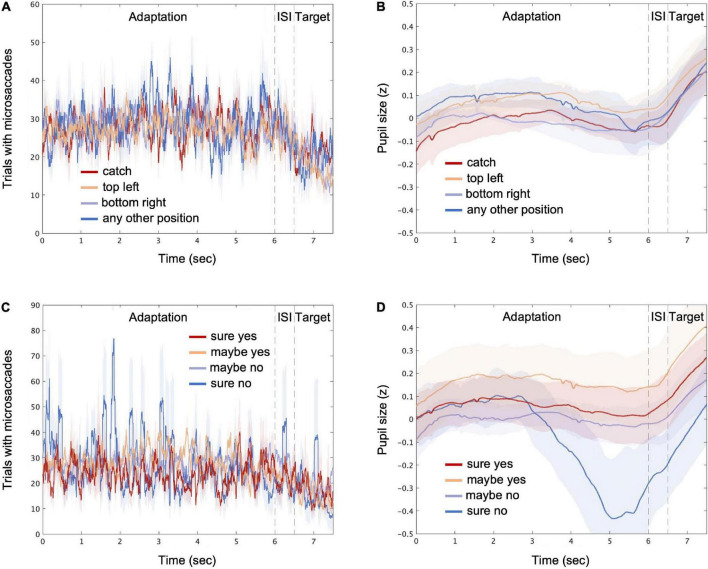
Microsaccades and pupil size over time. The average count of trials with microsaccades detected (see “Materials and methods”) is shown for stimulus conditions [“catch,” “top left,” “bottom right,” and “any other position”; **(A)**] and for response conditions [“sure yes,” “maybe yes,” “maybe no,” and “sure no”; **(C)**], respectively. The average pupil size is shown for different stimulus **(B)** and response **(D)** conditions, respectively. The time points consist of the adaptation, inter-stimulus interval (ISI), and target periods. The shaded areas represent ± 1 SE across all participants.

Figure 3 displays the transitions of the microsaccades and pupil size over time, each combined with the stimulus conditions (panels A and B) and the response conditions (panels C and D). The count of the trials with microsaccades fluctuated, and the pupil size, z-transformed within each participant, increased rapidly during the target presentation, which could be associated with the phasic locus coeruleus activity (Reimer et al., 2016; Breton-Provencher and Sur, 2019; Joshi and Gold, 2020). More specifically, for the “sure no” response condition, the microsaccades tended to burst intermittently, and the pupil size was seemingly reduced during the late phase of the adaptation, although the changes were not very large. Next, we took a machine learning approach to determine the possible links between perceptual decisions and the features derived from microsaccades and pupil size.

### Prediction of perceptual decisions from eye features

For each trial, the recorded eye movement and pupil size data were divided into the adaptation, ISI, and target periods. The means of the microsaccade rate and the pupil size were calculated for each period. The resulting six features (three time periods multiplied by two averaged eye behaviors) were sent to a sparse logistic regression classifier (Freedman, 2009) to decode the perceptual decisions about target presence. As vaguely suggested by the transitions of the microsaccades and pupil size over time (Figure 3), the peripheral responses during the periods of the adaptation and the ISI may possibly provide some information for decoding the subsequent perceptual decisions *via* changes in the degree of adaptation, prior expectation (e.g., Denison et al., 2011; Balcetis et al., 2012; Koizumi et al., 2019), and even internal states such as arousal and fatigue (e.g., Sheth and Pham, 2008; Lambourne and Tomporowski, 2010). Decoding was performed separately for each participant, and 10 × 10-fold stratified cross-validation (sCV) was performed. Accuracy and AUC were computed over the 100 folds to estimate the decoding performance. The sparsing parameter of the coefficient matrix was optimized to maximize AUC using a grid search. The statistical significance against the chance level was evaluated using a permutation test, in which the corresponding null distribution was estimated by permuting the labels in the data.

**FIGURE 4:**
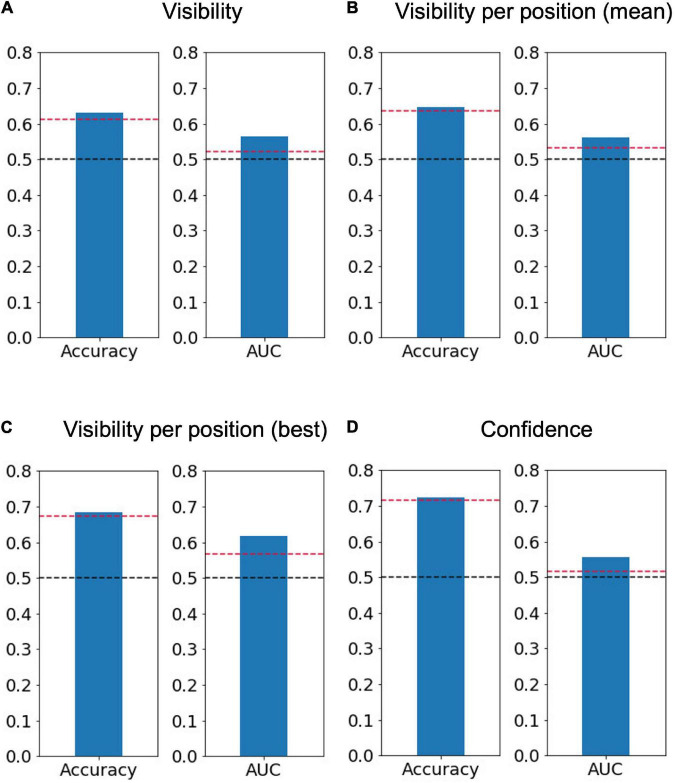
Average decoding accuracy and area under the receiver operating characteristic curve (AUC). Averaged accuracy across participants of **(A)** the perceptual-decision (“yes” vs. “no”) decoding calculated using both positions of interest (*N* = 7), **(B)** that calculated separately for the two positions of interest and then averaged (*N* = 7), **(C)** that for the best position when the trials were separated based on the target position (*N* = 7, top left for three participants and bottom right for four participants), and **(D)** confidence (“sure” vs. “maybe”) decoding (*N* = 6). The dashed red lines represent a significance level of 0.05 based on the permutation test, while the dashed black lines represent 0.5.

We successfully decoded the perceptual decisions about the target presence shown either at the top-left or bottom-right positions significantly above chance level, supported by BF_10_ indicating moderate evidence regarding AUC (accuracy = 0.63 ± 0.03, p = 0.003, BF_10_ = 9.54, AUC = 0.56 ± 0.02, *p* < 0.001, BF_10_ = 3.23, Figure 4A). The imbalance between two classes might affect the decoding accuracy; but the AUC, which is robust against response biases, was significantly higher than chance. Furthermore, even if the number of trials was equated by randomly resampling the visible trials, the AUC was significantly higher than chance (0.60 ± 0.01, *p* < 0.001, BF_10_ = 131.77). We also performed the perceptual-decision decoding separately for each of the two target positions (Figure 4B) and observed that the performance averaged across the two positions was also significantly above chance (accuracy = 0.64 ± 0.03, p = 0.04, BF_10_ = 8.21, AUC = 0.56 ± 0.02, *p* < 0.001, BF_10_ = 2.09) and was very similar to the decoder constructed from the data of both stimulus positions [t(6) = −0.26, *p* = 0.80, BF_10_ = 0.38 for accuracy and t(6) = 0.46, *p* = 0.66, BF_10_ = 0.79 for AUC]. The decoding performance was even higher when only the position with the best decoding performance was considered for each participant (accuracy = 0.68 ± 0.03, p = 0.02, BF_10_ = 18.20, AUC = 0.62 ± 0.03, *p* < 0.001, BF_10_ = 5.67, Figure 4C). Choosing the best position for each participant can be advantageous for achieving better decoding accuracy during future use of our method in neuroimaging experiments.

**FIGURE 5:**
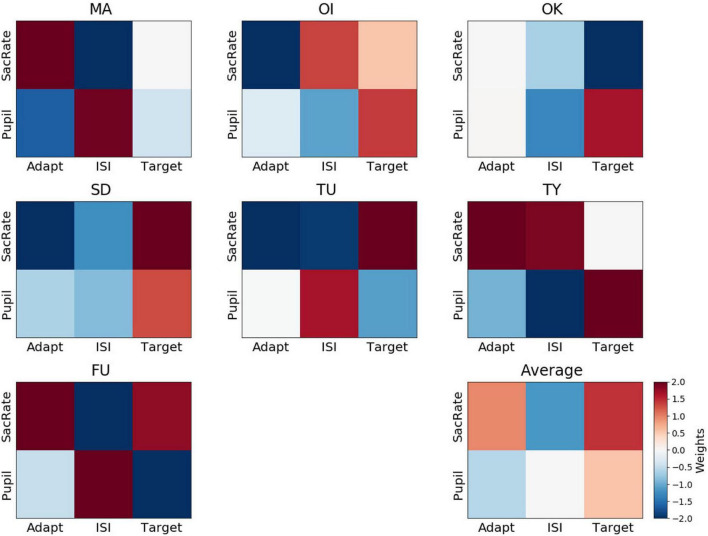
Weights assigned by the perceptual-decision decoder. Representation of decoder weights from every participant attributed to the mean microsaccade rate (SacRate) and the mean pupil size (Pupil) during the adaptation (Adapt), inter-stimulus interval (ISI), and target (Target) periods, respectively. For visualization purposes, the color scale was bounded between –2 and 2, but some weights were outside this range.

To understand which of the six features are critical for perceptual-decision decoding, we looked at the weights attributed to each feature by the decoders. Figure 5 shows the weights of each participant and their average. We found that all of the weights averaged across participants (lower-right panel) were not significantly different from 0, with BF_10_ values indicating anecdotal evidence or even weak support for the null hypothesis (BF_10_ = 0.38, BF_10_ = 0.54, BF_10_ = 0.62, BF_10_ = 2.34, BF_10_ = 0.39, BF_10_ = 0.48 for the microsaccade rate during the adaptation, ISI, and target periods and for the pupil size during the adaptation, ISI, and target periods, respectively). These results indicate that the feature weights were inconsistent across participants. In other words, the perceptual decision about target presence was not associated with a specific type of eye movement. For example, more frequent microsaccades occurring during the adaptation period were associated with the “yes” responses (positive weights indicated by a reddish color in Figure 5) for some participants (e.g., MA, TY, and FU), possibly due to less adaptation originating from unstable fixation. However, this was not the case for the other participants. Additionally, a larger pupil size during the target period was associated with a “yes” response for some participants (OI, OK, SD, TY), possibly due to their greater attention (Hoeks and Levelt, 1993), although this was not the case for the other participants. We note that it is unlikely that the decoder was merely reflecting random noise in the eye movements because a permutation test on the averaged decoding accuracy indicated that the accuracy was indeed highly significant relative to chance. Instead, one interesting reason for the inconsistency of the feature weights (and sparsing parameters) across participants, though speculative, could be that the perceptual states were determined by combinations of several factors, such as degree of adaptation, attention, and alertness, interacting with each other in a state-dependent manner, resulting in different balances of the feature weights across participants.

Regarding the relation between the microsaccade rate and pupil size, their feature weights did not correlate across participants during the adaptation and target periods (Pearson’s *r* = −0.42, BF_10_ = 0.68 and Pearson’s *r* = −0.15, BF_10_ = 0.48, respectively) while they correlated only during the ISI period (Pearson’s *r* = −0.87, BF_10_ = 6.44). These results indicate partly similar but mostly distinct contributions of microsaccades and pupil size.

Although we did not use blinking as a feature for decoding, it is possible that blink frequency, during which the pupil data were linearly interpolated, is at least partly related to perceptual decisions. To test this possibility, we examined whether the total blink duration in each trial differed by its label (yes/no). However, we found no difference in blink duration between the “yes” and “no” trials [0.24 ± 0.07 s for the “yes” trials and 0.23 ± 0.07 s for the “no” trials, t(6) = 0.71, *p* = 0.50, BF_10_ = 0.43], suggesting that the blink rate had little effect on the decoding.

It is also possible that the decoding performance depends on occasional gaze movements away from the fixation location, as such gaze movements would weaken retinotopic adaptation and might be related to the occurrences of microsaccades. To test this possibility, we identified trials that included any time points with gazes of more than 0.8 deg away from the fixation location, estimated by linear interpolation throughout the adaptation, ISI, and target periods, and distinguished 25% (SD = 20%) of the trials in total. Even after excluding these trials, perceptual decisions about the target presence shown either at the top-left or bottom-right positions were decoded significantly above chance level, supported by BF_10_ indicating moderate evidence regarding the AUC (accuracy = 0.66 ± 0.03, *p* < 0.001, BF_10_ = 13.50, AUC = 0.61 ± 0.03, *p* < 0.001, BF_10_ = 6.12).

Finally, a perceptual-decision decoder was trained with the data from all the participants together to test whether the decoder could detect a latent pattern that was common across the participants. This decoder was not better than the chance level (accuracy = 0.56, *p* = 0.98, AUC = 0.50, *p* = 0.68) and was outperformed by the individual decoders, again supporting the idea that the features that are useful for perceptual-decision decoding vary across participants.

### Prediction of perceptual confidence from eye features

We applied the same decoding method to predict the reported confidence level (sure/maybe) (Figure 4D). The decoding accuracy of the confidence level was computed using visible-only trials, invisible-only trials, or both visible and invisible trials to investigate whether the decision boundary between the low and high levels of confidence is dependent on perceptual decisions about target presence. One out of the seven participants who reported “maybe” in only one trial was removed from the analysis. The results show that the confidence level can be decoded significantly above chance level using the permutation test but with BF_10_ indicating anecdotal evidence regarding AUC (accuracy = 0.73 ± 0.03, *p* = 0.008, BF_10_ = 14.44, AUC = 0.56 ± 0.03, *p* < 0.001, BF_10_ = 1.04). When the visible and invisible trials were analyzed separately, the decoding performance was found to be significant for the visible trials (accuracy = 0.72 ± 0.05, *p* < 0.001, BF_10_ = 4.80, AUC = 0.65 ± 0.05, *p* < 0.001, BF_10_ = 1.84) but not for the invisible trials (accuracy = 0.68 ± 0.04, *p* = 0.11, BF_10_ = 1.58, AUC = 0.53 ± 0.01, *p* = 0.20, BF_10_ = 0.67). As suggested by the BF_10_ values for AUC, the feasibility of the confidence decoding would not be very high.

**FIGURE 6:**
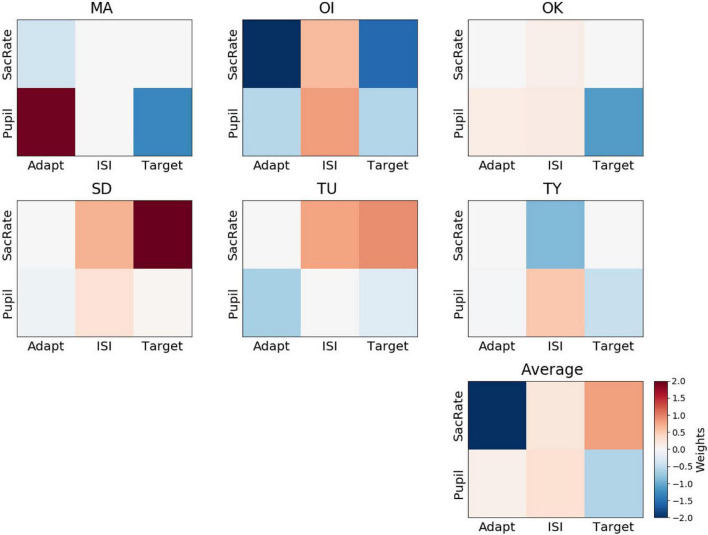
Weights of the perceptual-confidence decoder. Representation of decoder weights from every participant attributed to the mean microsaccade rate (SacRate) and the mean pupil size (Pupil) during the adaptation (Adapt), inter-stimulus interval (ISI), and target (Target) periods, respectively. For visualization purposes, the color scale was bounded between –2 and 2, but some weights were outside this range.

The feature weights for confidence were not consistent across participants (Figure 6), and none of the weights were significantly different from 0 (BF_10_ = 0.58, BF_10_ = 0.54, BF_10_ = 0.53, BF_10_ = 0.83, BF_10_ = 1.47, BF_10_ = 1.89 for the microsaccade rate during the adaptation, ISI, and target periods and for the pupil size during the adaptation, ISI, and target periods, respectively). For example, a larger pupil size during the ISI was associated with a “sure” response for some participants (OI, OK, SD, TY), possibly due to a higher amplitude of attention (Hoeks and Levelt, 1993), although this was not the case for the other participants.

When we compared the feature weights between the perceptual-decision decoding and the confidence decoding, the weight maps did not seem to overlap. On average, the correlation between the feature weights of the yes/no decoding and the confidence decoding was very low (Pearson’s *r* = 0.06 ± 0.27). This supports the dissociation of the mechanisms supporting Type 1 and Type 2 judgments, as has been suggested by recent neuroimaging data (Cortese et al., 2016; Peters et al., 2017).

## Discussion

In this experiment, eye movements and pupil sizes were recorded while participants performed a perceptual-decision task associated with the existence of the target stimulus in AIB. A sparse logistic regression classifier was trained on the features extracted from the microsaccade rate and pupil size data to verify whether they could be used to predict the subjective experience of the target stimulus on a trial-by-trial basis.

The decoding performance metrics, such as accuracy and AUC, suggested that it was possible to predict perceptual decisions about target presence at above chance level from the microsaccade rate and pupil size. The peripheral responses might be the trace of the neural processes that lead to the decisions or simply the result of the perceptual decisions. Although the present study is not designed to elucidate the causal links between perceptual decisions and peripheral responses, this could be tackled in future experiments by combining the current paradigm with the manipulation of peripheral responses possibly *via* biological feedback.

The moderate level of decoding accuracy can be partly explained by the fact that we used very weak visual stimuli to roughly equate the number of visible and invisible trials to build a decoder. Another possibility is that the perceptual-decision decoding for oblique target positions was more difficult than that for cardinal positions due to less typical oblique eye movements (e.g., microsaccades in oblique directions are relatively rare; Engbert, 2006). In future applications of this decoder in neuroimaging experiments, one may further optimize the predictability of perceptual decisions by selecting the target position that yielded a better decoding performance for each participant. The decoding was performed with the eye features simply averaged in each of the three periods to avoid overfitting due to a large number of interval parameters; however, using more finely grained intervals may also improve predictability partly because microsaccades and pupil size may be more informative at some time points than others (Strauch et al., 2021).

Interestingly, we found that the weight patterns were not consistent across participants (Figure 5), which explains why decoding performance decreased when all the participants’ data were merged to build a unitary classifier. Such an idiosyncrasy could be rather advantageous for future applications in neuroimaging experiments. Indeed, if the decoders are based on the peripheral responses in a consistent manner across participants, the NCC identified using a no-report paradigm might also reflect the neural responses related to the common peripheral responses. For example, if the “yes” trials are associated with more frequent microsaccades, the difference between the “yes” and “no” trials might include not only the neural responses that reflect the perceptual decisions but also those related to the microsaccades. However, we found that the decoder of each participant is based on different features of the microsaccade rate and pupil size (Figure 5). Therefore, if we average the difference in the neural responses between the “yes” and “no” trials across participants, the responses related to eye movements will be canceled out, and the average difference will mostly reflect the pure NCC.

Recent studies have already reported successful task-set decoding based on eye movements: A Naïve Bayes classifier with simple statistical features of eye fixation and saccades was able to recognize whether the participants were performing a picture memorization, searching, pseudo-reading, or reading task (Henderson et al., 2013). In a related study, the saccade amplitude and fixation features were measured in three different scene-viewing tasks (memorization, searching, or aesthetic preference; Kardan et al., 2015). While these procedures are highly useful, our current procedure should further advance the potential use of eye movements in neuroscience studies by enabling the trial-by-trial assessments of visual awareness for stationary targets in addition to moving targets (Frässle et al., 2014; Kapoor et al., 2022).

Since our decoder does not have perfect accuracy, in the context of a neuroimaging experiment, the inaccurately decoded trials could affect the average neural response of the perceptual decisions classes. As a result, the signal-to-noise ratio, namely the difference in the average neural response between the decisions, will be smaller in the current no-report paradigm than in a conventional report paradigm. To compensate for such a relatively low signal-to-noise ratio, the number of trials can be increased or the decoding accuracy can be improved by including other physiological responses, such as heartbeat rate and skin conductance response.

One concern associated with the use of this decoder in a neuroimaging experiment is that reporting itself might affect eye movements. That is, eye movements may change depending on whether the participants are required to report their percepts. In such cases, the accuracy of the decoder could drop during an applied neuroimaging session where participants do not engage in reporting. One way to validate whether the decoder is achieving sufficient accuracy during the neuroimaging session under the no-report paradigm is to compare the difference in the neural responses between the visible and invisible trials in both report and no-report paradigms. If the activation difference in the no-report paradigm is a subregion of that in the report paradigm (Frässle et al., 2014), we can claim that the decoder is reliable. In any case, a combination of report and no-report paradigms will be helpful for finding the true NCC (Tsuchiya et al., 2015) in future studies (e.g., Kapoor et al., 2022).

In the current study, we succeeded in inferring perceptual decisions during AIB by building sparse logistic regression classifiers based on microsaccade rate and pupil size. The important features for the decoder were not consistent across participants, which is advantageous in neuroimaging experiments because the neural response differences are not contaminated by the differences in eye movements. Nevertheless, the limitations of this study include the fact that the decoding accuracy was only moderately high and that the number of participants was limited; however, our decoding method is a valuable starting point for the design of a no-report paradigm for isolating the neural activities that facilitate perceptual awareness *per se*. Although we tested this method with AIB because of the advantages mentioned in the “Introduction” section, we believe it can be easily adapted to a variety of situations, such as binocular rivalry, bistable figures, or near-threshold stimuli. Certain critical points, including the generalization of the decoder across different recording sessions, should be addressed in future studies before using this decoder to identify the NCC in the no-report paradigm.

## Materials and methods

### Participants

Ten participants (three females) with normal or corrected-to-normal vision volunteered to take part in the experiment. Three participants were removed from the analysis because there were too many blink periods to reliably detect the microsaccade rate and pupil size (see below). All participants provided written informed consent to participate in this study, which was conducted in accordance with the ethical standards stated in the Declaration of Helsinki (2003) and approved by the local ethics and safety committees at the Center for Information and Neural Networks (CiNet), National Institute of Information and Communications Technology.

### Apparatus

The experiment was conducted in a dark room. A headrest was placed 50 cm away from the screen that displayed the visual stimuli to restrain head movements. As a result, the screen area was 40 deg in width and 30 deg in height with respect to the visual angle. The gaze position, eye velocity, acceleration, and pupil size of both eyes were recorded at a frequency of 500 Hz during the experiment using Eyelink 1000 (SR Research) placed 40 cm away from the participants. The display of the stimulus and control of the eye tracker were implemented using MATLAB Psychtoolbox (Brainard, 1997; Kleiner et al., 2007) and Eyelink Toolbox (Cornelissen et al., 2002).

### Stimulus and task

The recording session was divided into different blocks as described in the following “Procedure” section of the “Materials and methods.” In the first trial of each block, an adaptation stimulus was presented for 60 s. After an inter-stimulus interval of 0.5 s, the target stimulus was presented for 1 s (target period). Then, the response screen showing the key assignments was displayed until the participant pressed a button. In the subsequent trials, the adaptation stimulus was presented for only 6 s (adaptation period). An example of a trial timeline is shown in Figure 1.

The adaptation stimulus was composed of eight drifting Gabor patches; their centers were evenly spaced on a circle with a radius of 12.4 deg, each of which was on the cardinal and diagonal axes, respectively. Each grating contrast was filtered through a Gaussian envelope with standard deviation of 0.8 deg. During the adaptation period, the spatial phase of a flickering sinusoidal grating changed with a triangular wave. The spatial and flickering frequencies were 1.6 cycle/deg and 9.2 Hz, respectively. The fundamental frequency of the triangular wave was 0.83 Hz, which yielded grating drifting at 0.52 deg/s. The contrast of the sinusoidal grating was set to 200%; further, the luminance values smaller than 0 were set to be 0, while those larger than 255 were set to be 255. The four gratings on the horizontal and vertical axes moved in the opposite direction of the four gratings on the diagonals.

The target stimulus consisted of a static Gabor patch in which the contrast slowly increased and then decreased with a temporal Gaussian (SD = 166 ms) function. The peak contrast of the target stimulus was determined during a calibration experiment (see the “Procedure” section). If the target location is predictable, participants will pay attention to the target stimulus, which makes it difficult to make the target invisible. To make the prediction of the target location difficult, the target stimulus was presented in one of the eight locations where the adaptation stimulus was presented. In a pilot study where the target contrast was kept constant across all positions at around a threshold level, the reported visibility was not consistent across positions (e.g., 70% visible in one position, and 30% visible in another position). Because the visible and invisible responses appeared to have almost equal probability at the top-left and bottom-right positions consistently across participants in the pilot study, the target stimulus was presented at these positions (positions of interest) in 65% of the trials and at other locations in 15% of the trials in the main experiment. The remaining 20% of the trials were catch trials, where the target stimulus was absent. Although it was ideal to present stimuli in eight locations with equal probability, we presented the target more frequently at the two positions of interest to obtain enough data for training the decoders.

The participants were instructed to maintain fixation on a bullseye at the center of the screen and report if they saw the stimulus, along with their confidence level (“sure yes,” “sure no,” “maybe yes,” or “maybe no”) by pressing a button. They were asked to distribute their responses between the two levels of confidence. The key response assignments were displayed 0.5 s after the end of the target presentation around the fixation point in a 2 × 2 pattern (Figure 1). Four different colors (proximal to red, green, yellow, and purple) appeared in the quadrants around the fixation point to help participants recognize the locations of the assigned keys with the same color labels. The answers in the same row shared the same perceptual decision (“yes” or “no”), while those in the same column shared the same confidence level (“sure” or “maybe”). There were four possible arrangements, and these were used in a random order for each block.

### Procedure

The experiment was divided into two parts: the calibration and the main experiment. In the calibration part, the contrast threshold of the target stimulus (peak of the temporal Gaussian) was determined using a fixed-step staircase method (one up and one down rule, 0.05 log unit steps). Calibration was stopped after 15 inversions of the staircase or 65 trials. The contrast threshold was defined by the average of all the reversal values except the first two, which can bias the estimate (García-Pérez, 1998). Only responses to the positions of interest were taken into account during the calibration.

In the main part of the experiment, the target stimulus contrast was fixed at the threshold estimated during calibration. The main experiment consisted of four blocks of 43 trials. Between each block, the participants could rest if they wanted. The key assignment for the behavioral response was fixed within each block, and all participants went through every four-key arrangement in a random order. Because the decoders were built from the data across all four blocks with different key assignments, they were insensitive to a specific key assignment for the combination of perceptual decisions and confidence ratings. The calibration of the eye-tracker was conducted before each block.

### Preprocessing

To assess the quality of the recordings, the blink events contaminating the data were identified. These events were defined as the periods from 200 ms before to 200 ms after the time point when the pupil size measurements for both eyes were missing. Partial blink events, where the pupil was not fully occluded, were identified as the period from 200 ms before to 200 ms after the time point when the pupil size varied more than 20 arbitrary units per sample (approximately 0.5 mm^2^; Troncoso et al., 2008).

The pupil size at each time point was calculated in the following way: If the pupil size for both eyes was available, they were averaged. If the pupil size data from only one eye were available, they were used directly as the pupil size measure. Finally, if no pupil data were available, they were estimated by linear interpolation. Three participants were removed from the experiment because more than 45% of their data were interpolated (Smallwood et al., 2011); thus, their pupil size estimation was not very reliable, and the microsaccade data were missing for this period. Finally, the data from the remaining seven participants were filtered by a low-pass filter (10 Hz cutoff), and the pupil size was z-transformed within participants over the adaptation, ISI, and target periods (Smallwood et al., 2011).

Microsaccade detection was performed using an unsupervised clustering method, which has been fully described in a previous study (Otero-Millan et al., 2014). Briefly, for each eye, velocity was computed from the horizontal and vertical instantaneous eye velocity $v=\sqrt{v_{\text{x}}^{2}+{\text{v}}_{\text{y}}^{2}}$ and averaged for the two eyes, and acceleration was processed similarly. The microsaccade instances were identified by selecting the six highest velocity peaks per second to include all the true microsaccades. The maximum velocity peak, initial acceleration, and final acceleration of each candidate were extracted and z-transformed across the microsaccade instances. A principal component analysis was performed, and the components with eigenvalues larger than 5% of the maximum were kept. Next, K-mean clustering (*K* = 2) was applied to separate the noise from the microsaccades. The cluster with the largest average peak velocity was identified as the cluster of microsaccades. Finally, we checked whether the microsaccades satisfied the following conditions: a minimum duration of 8 ms (Leopold and Logothetis, 1998), amplitude between 10 and 120 min of arc, and mean velocity between 3 deg/s and 120 deg/s (Martinez-Conde et al., 2004). As a quality check, the correlation coefficient between the peak velocity and the amplitude of the microsaccades was computed. A correlation of higher than 0.6 was found for every participant, which satisfied the “main sequence” criterion (Zuber et al., 1965). The microsaccade rate was defined by the number of detected microsaccades divided by the duration of each period (adaptation, ISI, and target). All the preprocessing procedures were implemented in Python 2.

### Perceptual-decision decoding

To test whether the perceptual decisions regarding target presence can be determined from the extracted features, a sparse logistic regression decoder was trained for each participant.

The decoding was performed using data from the two target positions of interest (56 trials per position). From each trial, six features were extracted corresponding to the mean of the microsaccade rate and the pupil size calculated for each period of the trial (the adaptation, inter-stimulus interval, and target period).

Given the small number of trials per participant, the decoding performance was estimated using a 10-fold stratified cross-validation (sCV). The data were standardized, and the reported decision (“yes” vs. “no”) of the test fold was decoded. Automatic pruning of the decoder weights was performed with L1-regularization. The sparsing parameter (C) was optimized using a grid search (31 values between 1 and 103 that are evenly spaced on a logarithmic scale). In our case, a small value of C resulted in a sparser weights matrix. The decoding performance was assessed by computing the accuracy and the AUC over the 100 folds.

For the statistical analysis, we performed decoding assessments with 1,000 permutations of the dataset. For each permutation, the decoding accuracy for each participant was calculated after the trials corresponding to a “yes” response were selected randomly, and the accuracy was averaged across participants. The statistical significance against the chance level was evaluated by comparing the original averaged decoding accuracy with the permutation distribution of the averaged decoding accuracy.

For the corroborating analysis, we also tested the null hypotheses that accuracy, AUC, and weight are not different from the chance levels, with the Bayesian paired samples *t*-test provided in the JASP statistical software (JASP Team, 2019). BF_10_ is the ratio of the likelihood of the data under the alternative hypothesis divided by that under the null hypothesis. Values larger than 1 indicate that the data favor the alternative hypothesis over the null hypothesis (1–3: anecdotal, 3–10: moderate, over 10: strong evidence in support of the alternative hypothesis), while values smaller than 1 indicate the opposite (e.g., Wagenmakers et al., 2016).

For the decoding using the data from all the participants, we used all four-block data (including all four-key assignments) of six participants as a training set and all four-block data of one other participant as a test set, which were repeated for all seven combinations.

All the machine learning computations were carried out in Python using the scikit-learn module (Pedregosa et al., 2011).

### Confidence decoding

We also built the confidence decoder. The procedure was the same as the perceptual-decision decoder, except that the label to be classified was a confidence rating (low vs. high). The confidence decoding was performed for the only “yes” trials, only “no” trials, and both “yes” and “no” trials. The statistical significance was evaluated by the permutation test and the Bayesian paired samples *t*-test, which were also used for the perceptual-decision decoding.

## Data availability statement

The raw data supporting the conclusions of this article will be made available by the authors, without undue reservation.

## Ethics statement

The studies involving human participants were reviewed and approved by the Ethics Committee of the National Institute of Information and Communications Technology. The patients/participants provided their written informed consent to participate in this study.

## Author contributions

KA and IM designed the experiment. J-BB performed the experiment. J-BB and RN analyzed the data. All authors contributed to the article and approved the submitted version.
