# Supplementary material for: Building a decoder of perceptual decisions from microsaccades and pupil size
Source: Front Psychol. 2022 Sep 13;13:942859. doi: 10.3389/fpsyg.2022.942859 (PMC9514321; doi:10.3389/fpsyg.2022.942859)
Supplement: Supplementary file 1 [file Data_Sheet_1.pdf]

# Supplementary material

## Prediction of stimulus position from eye features

We also built the stimulus position decoder. The procedure was the same as the perceptual-decision decoder, except that the label to be classified was stimulus position (top left vs. bottom right). The statistical significance was evaluated by the permutation test and the Bayesian paired samples t-test, which were also used for the perceptual-decision decoding.

Given that none of the data (e.g., microsaccade rate) reflected the directionality of eye movement, it is predicted that the target position cannot be decoded from such data. However, we found that the decoding accuracy was relatively low but significant, supported by $BF_{10}$ indicating moderate evidence regarding AUC (accuracy = 0.54 ± 0.01, $p = 0.034$, $BF_{10} = 4.78$, AUC = 0.54 ± 0.01, $p = 0.034$, $BF_{10} = 3.40$, Figure S1). This would suggest that non-directional measures of eye movement (microsaccade rate and pupil size) might provide some information about the stimulus position.

The feature weights for position decoding were not consistent across all participants (Figure S2). We found that all of the weights averaged across participants (lower-right panel) were not significantly different from 0 ($BF_{10} = 0.42$, $BF_{10} = 0.45$, $BF_{10} = 0.74$, $BF_{10} = 0.55$, $BF_{10} = 0.41$, $BF_{10} = 1.26$ for the microsaccade rate during the adaptation, ISI, and target periods and for the pupil size during the adaptation, ISI, and target periods, respectively).

Given that the detection performance interacted with the target position and that the target position can be decoded, one may consider the possibility that the perceptual-decision decoding reflected information about the target position rather than the perceptual decision itself. However, this is unlikely because the perceptual-decision-decoding accuracy and AUC remained significant even when we analyzed the data separately for each target position (Figure 4B). At least the perceptual-decision decoding was not entirely the same as the position decoding, as the former outperformed the latter.

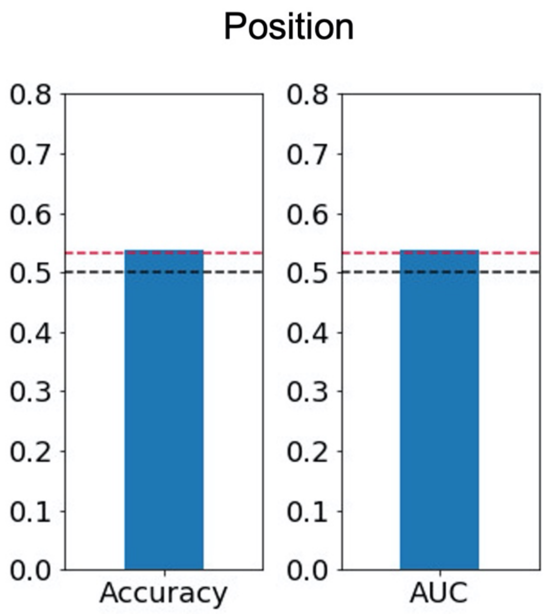


*Figure S1. Average decoding accuracy and area under the receiver operating characteristic curve (AUC). Averaged accuracy across participants of target-position ("top left" vs. "bottom right") decoding (N = 7). The dashed red lines represent a significance level of 0.05 based on the permutation test, while the dashed black lines represent 0.5.*

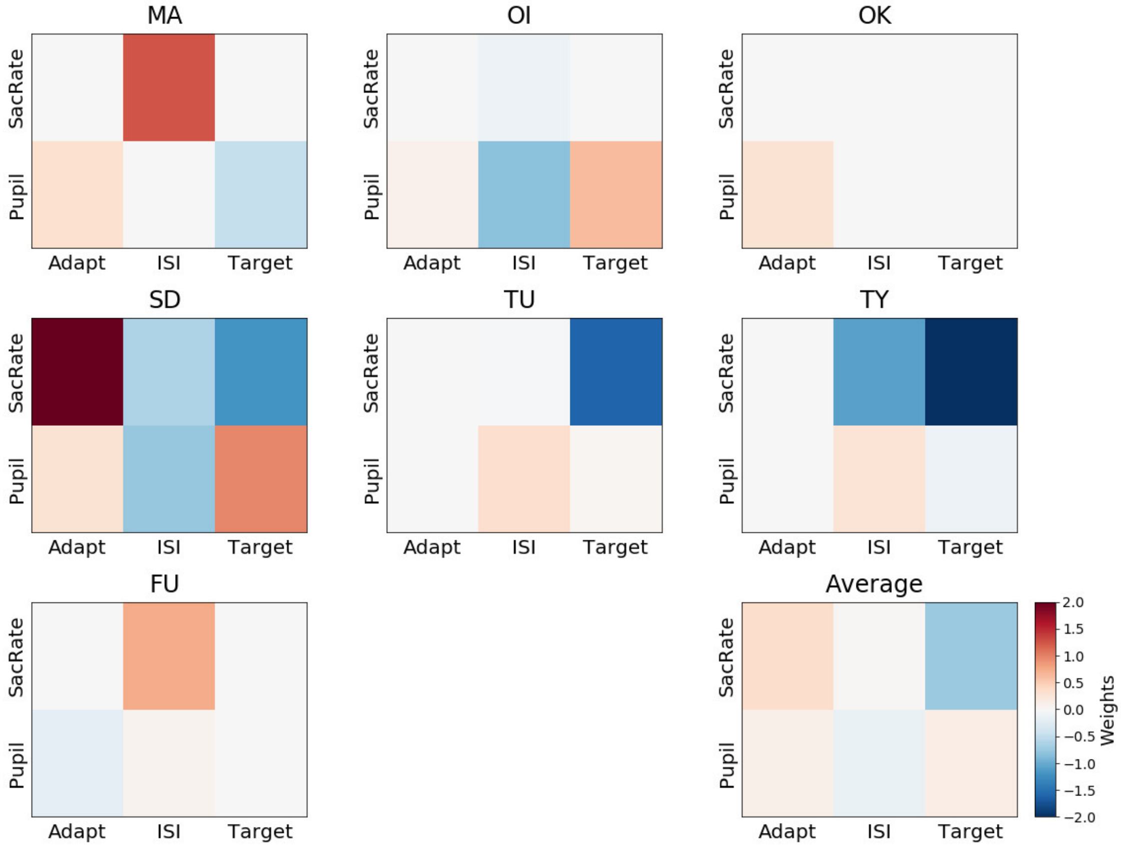


*Figure S2. Weights of position decoder. Representation of decoder weights from every participant attributed to the mean microsaccade rate (SacRate) and mean pupil size (Pupil) in the adaptation (Adapt), inter-stimulus interval (ISI), and target (Target) periods, respectively. For visualization purposes, the color scale was bounded between -2 and 2, but some weights were outside this range.*
